# Weakened snow and ice melting by enhanced cloud short-wave cooling effect in the Arctic

**DOI:** 10.1093/nsr/nwaf116

**Published:** 2025-03-27

**Authors:** Annan Chen, Chuanfeng Zhao, Haotian Zhang, Yikun Yang, Jing Li, Yan Yu, Qinghong Zhang, Jiefeng Li

**Affiliations:** Department of Atmospheric and Oceanic Sciences, School of Physics, Peking University, Beijing 100871, China; Department of Atmospheric and Oceanic Sciences, School of Physics, Peking University, Beijing 100871, China; Department of Atmospheric and Oceanic Sciences, School of Physics, Peking University, Beijing 100871, China; Department of Atmospheric and Oceanic Sciences, School of Physics, Peking University, Beijing 100871, China; Department of Atmospheric and Oceanic Sciences, School of Physics, Peking University, Beijing 100871, China; Department of Atmospheric and Oceanic Sciences, School of Physics, Peking University, Beijing 100871, China; Department of Atmospheric and Oceanic Sciences, School of Physics, Peking University, Beijing 100871, China; Department of Atmospheric and Oceanic Sciences, School of Physics, Peking University, Beijing 100871, China

**Keywords:** cloud radiative effect, surface albedo, snow/ice coverage

## Abstract

Clouds and surface albedo significantly affect the energy balance on Earth. With the snow/ice melting in the Arctic, an understanding of how this impacts short-wave cloud radiative effects (CRE) remains a critical, yet poorly understood, question. We analysed snow/ice coverage (SIC) changes and their impact on the CRE in the Arctic from the past to the present and to future scenarios. From 2000 to 2020, the SIC decreased by –0.058/decade, leading to CRE changes (∆CRE) at the top of the atmosphere and surface by –1.25 ± 0.49 and –0.21 ± 0.20 W/m²/decade, with an average reduction in the sea ice melting rate (${\mathrm{\Delta }}\mathcal{R}$) of 3.45 cm/year. The sea ice coverage and the CRE remained relatively stable from 1850 to 1915, followed by dramatic declines in both sea ice coverage and CRE under both 245 and 585 shared socio-economic pathways post-2015. The snow/ice loss amplifies the cloud-induced short-wave cooling effect, partly slowing further melting yet not preventing overall snow/ice loss.

## Teaser

The increased snow/ice loss amplifies cloud short-wave cooling effects, therefore turns out to be mitigating further melting partly.

## INTRODUCTION

**Figure 1.**
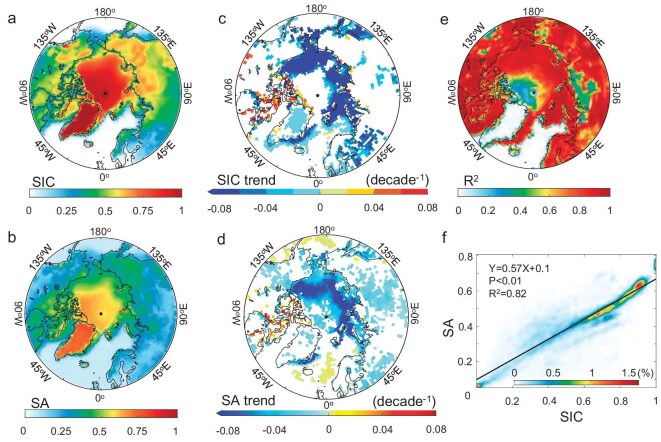
Spatial and temporal distribution of the SIC and the SA, along with their linear relationship. (a, b) Spatial distribution of the multi-year average of (a) the SIC and (b) the SA at 60°N to 90°N from March 2000 to February 2020. (c, d) Long-term trends of (c) the SIC and (d) the SA at 60°N to 90°N from March 2000 to February 2020, with blank areas indicating that *P* > 0.05. (e) Spatial distribution of goodness of linear fitting between the SIC and the SA. (f) Probability distribution of the SIC and the SA in the grids in which both the SIC and the SA show significant trends, along with the linear fitting between the SIC and the SA represented by the black line.

The near-surface temperature over the Arctic has undergone a warming rate that is two to nearly four times faster than the global average—a phenomenon termed Arctic Amplification [1,2]. The causes of Arctic Amplification have been attributed to various mechanisms; however, their relative importance remains under debate [3]. Among the various mechanisms, ice–snow albedo feedback is considered one of the primary drivers during the warm season [4,5]. This feedback mechanism indicates that elevated surface temperatures contribute to a reduction in the snow/ice coverage (SIC), which is then replaced by either water or land. The SIC represents the proportion of the spatial extent of snow- and ice-covered surfaces in a given region or grid. Consequently, there is a decrease in the surface albedo (SA), resulting in increased absorption of short-wave radiation [6]. This leads to further warming and a more significant loss of the reflective components of the cryosphere [7]. Snow and ice play a vital role in the energy balance of Earth due to their higher albedo compared with any other natural surfaces [8]. In the Arctic, the SA is mainly regulated by features of snow and ice such as the SIC and ice-surface characteristics. For example, this study suggests that a 1% change in the SIC results in an ∼0.57% change in the SA on average in the Arctic, as shown in Fig. 1.

Clouds regulate the radiative flux at the top of the atmosphere (TOA) and the surface (SFC) by altering the radiative transfer process, which further affects the melting and formation of snow and ice [9–11]. Although knowledge and understanding of the processes that determine Arctic Amplification have been improving swiftly [5,12], the high SA, frequent temperature and moisture inversion, low temperature and humidity, and the absence of sunlight for half of the year lead to the interaction between clouds and radiation becoming particularly complex in the Arctic [13,14]. The numerous interactions of physical processes that cause Arctic Amplification are intertwined and difficult to partition. For example, the increase in the spring cloud amount could balance the increase in the surface temperature or decrease in the SA [15]. Clouds decrease short-wave radiation that reaches the surface but increase the downward long-wave radiation at the surface. Climate models are required to quantify the individual contributions of feedback processes to Arctic climate change [16]. However, due to complex and sensitive feedback processes, Arctic climate modeling is extremely challenging [17]. Many climate simulations struggle to replicate historical features of the Arctic, such as the probability density function, spectra and mean spatial pattern of precipitation, clouds and sea ice [18]. The difference in simulation results across various models is large, suggesting that some important processes are either inadequately described or not included [19–21]. Consequently, reliable predictions of the future evolution of the Arctic climate remain elusive [22].

A critical issue in the study of Arctic climate change is an understanding of the interactions between clouds, surface and radiation, and how they collectively contribute to Arctic Amplification. Due to the rapid melting of ice and snow, sea ice cover at the end of summer has halved in the past 40 years and is projected to become ice-free by mid-century, potentially as early as the 2030s, in the Arctic Ocean [23,24]. Therefore, there is an urgent question regarding the way in which Arctic clouds are affected by changes in Arctic snow/ice characteristics through radiative processes. Although the interaction between clouds and long-wave radiation is important in the energy balance over the Arctic [9], in this article, we focus on the influence of clouds on short-wave radiation. In previous studies, Donohoe and Battisti [25] decomposed the contribution of atmosphere and surface to the albedo at the TOA, followed by Datseris and Stevens [26] and Hadas *et al.* [27], who quantified the contribution of clouds to the TOA albedo. The surface contribution to the TOA albedo has also been estimated [28]. Besides, the differential response of the TOA radiative fluxes to incremental changes in different variables, including the SA, is quantified by an effective method termed the ‘radiative kernel’ [29,30]. However, the impact of the surface on the atmospheric system transmittance, which directly affects short-wave radiation and the formation/deformation of snow/ice at the surface, is rarely quantified.

To fill this gap, we analyse the SIC changes during the period from 2000 to 2020 and their impact on the cloud short-wave radiative effect (CRE) at both the surface and the TOA from satellite data recordings. The linear relationship between the SIC and the SA is established in each grid at 60°N to 90°N [31,32] and the change in the CRE (∆CRE) due to the change in the SIC over a certain period is estimated. Moreover, we analyse the temporal dynamics of the sea ice coverage, CRE and ∆CRE from a historical period (1850–2015) to future scenarios (2015–2100) under two shared socio-economic pathways (SSPs), by utilizing data from state-of-the-art climate models from the Coupled Model Inter-comparison Project Phase 6 (CMIP6). To our knowledge, this study uniquely assesses the ∆CRE and its impact on the SIC in the Arctic, marking the analysis that integrates satellite observations with CMIP6 model outputs.

## RESULTS

### The spatial and temporal distribution of surface and cloud properties

A Moderate Resolution Imaging Spectroradiometer (MODIS) and the Clouds and the Earth's Radiant Energy System (CERES) provide cloud and radiative flux data from 2000 to the present. To maximize the data-coverage period and avoid the impact of major social events such as the COVID-19 pandemic that emerged in 2020 [33], this study focuses exclusively on the period from 2000 to 2020. From March 2000 to February 2020, the central Arctic Ocean and regions near Greenland exhibited the highest SIC, with averages surpassing 0.8. In the Greenland Sea and the Barents Sea, the SIC is observed to be the lowest—generally <0.2 (Fig. 1a). Figure 1b is similar to Fig. 1a, but it shows the spatial distribution of the multi-year average of the SA. The spatial distribution of the SA positively corresponds to the SIC—that is, a higher SIC is associated with a higher SA. Regions in Greenland and the central Arctic Ocean exhibit a high SA, with values of >0.5, whereas the Greenland Sea and the Barents Sea have notably lower SA values. The long-term trends in snow and ice characteristics, along with cloud and radiative features, are evaluated by using the Theil–Sen Median trend estimation and the Mann–Kendall trend test. Figure 1c depicts the long-term SIC trends in the Arctic from March 2000 to February 2020, highlighting significant declines along the Beaufort, Chukchi, East Siberian, Laptev and Kara Seas and in parts of the Greenland Sea, with the most pronounced declines surpassing –0.08/decade. Notable decreases in the SA in some areas align with the SIC trends (Fig. 1d). The averages of the SIC and the SA in the region of 60°N to 90°N demonstrate a significant decreasing trend, with the SIC at –0.016/decade and the SA at –0.022/decade (*P* < 0.01). The goodness of fit between the SIC and the SA is >0.8 in most of the Arctic region, which indicates that, in the Arctic, the SIC is one of the important factors that influence the SA, as shown in Fig. 1e. In these grids, in which the SIC and the SA both show significant changes, there is a good linear relationship between the SIC and the SA, as shown in Fig. 1f, and every 1% change in the SIC causes ∼0.57% changes in the SA on average.

During the same period, the Greenland Sea and the Barents Sea have a high cloud fraction (CF) multi-year average, surpassing 0.8. The central Arctic regions exhibit a slightly lower CF, of between 0.7 and 0.8, and the lowest CF is on the land, especially in northern Greenland, with values of <0.5 (Fig. S1a). The multi-year average of the cloud optical depth (COD) is similar to the CF, as shown in Fig. S1b. The COD is higher in the Greenland Sea and the Barents Sea, with values of >5, whereas it is slightly lower in the central Arctic, with values of ∼4.5. Figure S1c and d shows the long-term trends of the CF and COD, respectively. The region with a significant increase in the CF is located in the northern part of the Eurasian continent, with the maximum approaching 0.05/decade. The region with significant decreasing trends in the CF is located in the Greenland Sea, whereas the COD shows a significant downward trend in these two regions. However, in the regions in which the SIC has significantly decreased, including the Beaufort, Chukchi, East Siberian, Laptev and Kara Seas, as well as some parts of the Greenland Sea, the CF and COD do not show significant long-term trends, which is worthy of further investigation in the future, and the lack of significant long-term trends of the CF and COD highlights the impact of the SIC or the SA on the CRE studied here.

### The variations in CRE resulting from changes in SIC

**Figure 2.**
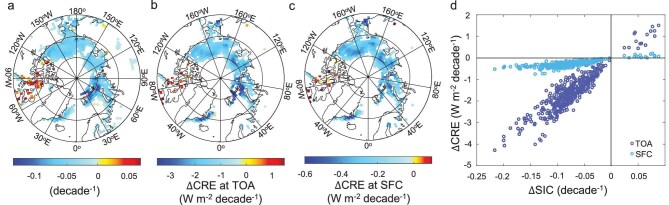
Spatial distribution of changes in short-wave radiative effect of clouds (∆CRE) at both the TOA and the SFC, and the SA due to a change in the snow/ice coverage (∆SIC), as well as the statistical relationship between ∆CRE and ∆SIC. (a) Changes in the SA due to ∆SIC from March 2000 to February 2020 at 60°N to 90°N, with shaded grids representing the points with the linear fitting between the SIC and the SA passing *P* < 0.05 and a goodness of fit of >0.8° (b, c) Spatial distribution of ∆CRE at the (b) TOA and (c) at the SFC. (d) Scatter plots between ∆SIC and ∆CRE at the TOA and the SFC.

By utilizing the linear relationships between the SIC and the SA, and the functions between the CRE and the SA for each grid in the annual average, the ∆CRE due to changes in the SIC (∆SIC) can be quantified: a 0.01 variation in the SIC is associated with a change of ∼0.29 W/m² in the CRE at the TOA and 0.05 W/m² at the SFC. Only grids that satisfy a goodness of fit between the SIC and the SA of >0.8 and *P* < 0.05 are selected and analysed in Fig. 2. Figure 2a shows the spatial distribution of the SA changes caused by ∆SIC in each grid from March 2000 to February 2020, which is calculated by using Equation (1) in the Methods section. The linear-fitting slopes between the SIC and the SA in the selected grids lie between 0.6 and 0.8, also suggesting that SA trends align with the changing pattern of the SIC. The decline rate of the average SIC in the selected grids is –0.058/decade, causing ∆CRE to reach –1.25 ± 0.49 and –0.21 ± 0.20 Wm^2^/decade at the TOA and the SFC (Fig. 2b and c), respectively. Figure 2d shows the scatter plots between ∆CRE and ∆SIC in the selected grids, which reveal that ∆CRE is positively correlated with ∆SIC at both the TOA and the SFC. In the first quadrant, as ∆SIC increases, ∆CRE is positive whereas, in the fourth quadrant, a decrease in ∆SIC is associated with a negative ∆CRE. Physically, with the SA decline, the TOA albedo and the atmospheric system transmittance decrease under both the all-sky and the clear-sky conditions, as shown in Equations (2) and (3) in the Methods section. However, cloud mitigates the TOA albedo reduction but accelerates the atmospheric system transmittance decrease, resulting in ${R_{clr}} - {R_{all}}$ and ${T_{all}} - {T_{clr}}$ being more negative (Fig. S2), and thus exerting a colder short-wave radiative effect.

### Enhanced cloud short-wave cooling effect mitigates snow/ice melting in the Arctic

**Figure 3.**
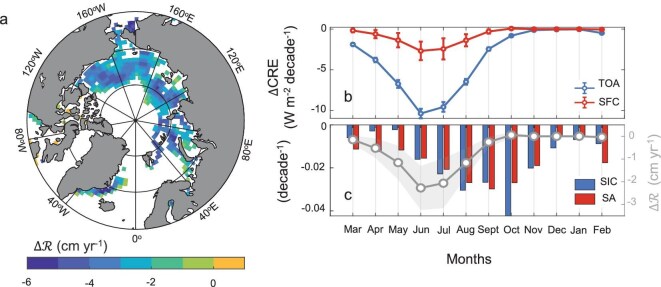
Spatial and temporal distribution of the sea ice melting rate change (${\mathrm{\Delta }}\mathcal{R}$). (a) Geographical distribution of the annual average of ${\mathrm{\Delta }}\mathcal{R}$. (b) Monthly variations of ∆CRE at the TOA and the SFC, with the error bars representing the standard deviation. (c) Monthly average trends of the SIC and the SA (histogram) and the variation in ${\mathrm{\Delta }}\mathcal{R}$ (gray line), with the shadow representing the standard deviation of ${\mathrm{\Delta }}\mathcal{R}$.

Considering that the decline in the SIC and the SA is mainly located in the marginal waters of the Arctic Ocean, such as the Beaufort, Chukchi, East Siberian, Laptev and Kara Seas, as well as parts of the Greenland Sea, Fig. 3a presents the spatial distribution of ${\mathrm{\Delta }}\mathcal{R}$ only in these regions. The average ${\mathrm{\Delta }}\mathcal{R}$ is ∼3.45 cm/year, suggesting that the ∆CRE could potentially slow down the sea ice-depth decline by an average of ≤3.45 cm/year. The ${\mathrm{\Delta }}\mathcal{R}$ is mainly located in the Beaufort, Chukchi, East Siberian, Laptev and Kara Seas and in parts of the Greenland Sea, with a maximum value reaching 10 cm/year. Because the SIC and the SA show significant decreasing trends (*P* < 0.05), with the SA showing the largest decrease in September (–0.035/decade) and the SIC showing the largest decrease in October (–0.048/decade), as shown in Fig. 3c, the ∆CRE is negative in all months (Fig. 3b). However, there is an obvious inner-annual cycle of ∆CRE, with the most negative value reached between June and July at both the SFC and the TOA. Quantitatively, the most negative values of ∆CRE are –10.37 ± 0.59 and –2.67 ± 1.14 W/m^2^/decade at the TOA and SFC, respectively. Besides, ${\mathrm{\Delta }}\mathcal{R}$ also shows an obvious monthly cycle, which is similar to that of ∆CRE, with the most negative value being –2.27 ± 0.96 cm/year. The peak time for ∆CRE or ${\mathrm{\Delta }}\mathcal{R}$ occurs in June and July, attributable to the combination of maximum insolation and a relatively higher decline in sea ice during these months whereas the peak time of ∆SA or ∆SIC is in September or October, driven by the release of ocean heat in the colder season [3,34].

It should be noted that, with variations in Arctic snow and ice, cloud properties are anticipated to adjust accordingly, thereby impacting the formation/deformation of snow and ice and altering the SA. The contribution of other factors besides the SA or the SIC to the CRE (${\mathrm{\Delta }}CR{E_{{total}}} - \Delta CRE$) and corresponding ${\mathrm{\Delta }}\mathcal{R}$ is also evaluated as shown in Fig. S3. The multi-year averages ‘${\mathrm{\Delta }}CR{E_{{total}}} - \Delta CRE$’ are –2.85 ± 1.35 and –3.46 ± 1.63 W/m^2^/decade at the TOA and SFC with similar interannual cycles to ∆CRE, suggesting that the most negative values of –12.20 ± 0.95 (SFC) and –9.28 ± 0.68 (TOA) W/m^2^/decade occur in June. ${\mathrm{\Delta }}\mathcal{R}$ also reaches the most negative value (–10.34 ± 8.00 cm/year) in June. ‘${\mathrm{\Delta }}CR{E_{{total}}} - \Delta CRE$’ at the SFC is more negative than ${\mathrm{\Delta }}CR{E_{sfc}}$, suggesting that the CRE are contributed by other factors that exert a colder effect on the snow and ice cover. However, ${\mathrm{\Delta }}CR{E_{toa}}$ is more negative than ${\mathrm{\Delta }}CR{E_{sfc}}$ whereas ‘${\mathrm{\Delta }}CR{E_{\textit{total}}} - \Delta CRE$’ is not, which likely suggests that the melting of the SIC declines the atmospheric absorption of short-wave-radiation by weakening the multiple reflection between the cloud and the surface [25].

### Enhanced cloud radiative cooling effect in the future climate

**Figure 4.**
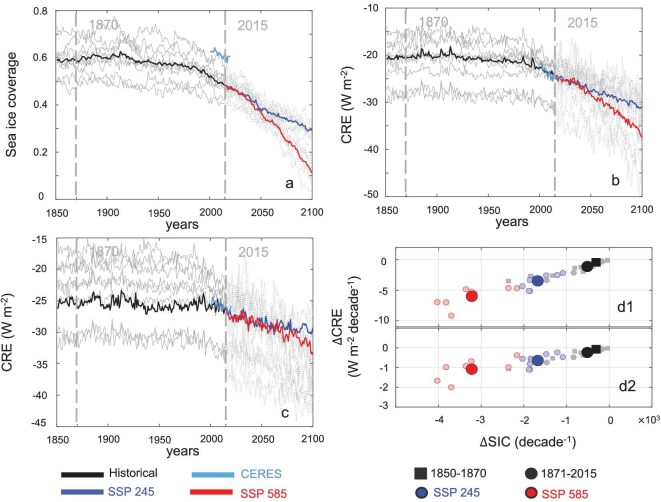
Sea ice coverage (a) and CRE at the TOA (b) and SFC (c) changes in Coupled Model Inter-comparison Project Phase 6 (CMIP6) runs of future scenarios. The gray lines show the historical and two SSPs time series of nine models. (d) Scatter plots between the change in the SIC (∆SIC) and the change in the CRE (∆CRE) at the TOA (d1) and the SFC (d2) in the historical and future scenarios. The black square and circle represent the multi-models mean in the historical periods; the blue and red circles represent the multi-models mean in SSP 245 and SSP 585 scenarios; and the light color represents the mean values of each model in specific periods.

As the results show above, the CRE from CERES are more negative from 2000 to 2020 due to melting snow and ice coverage. What about the future? To answer this question, we analyse the outputs of nine models that participated in the CMIP6 listed in Table S1. Figure 4 presents the time series of sea ice coverage and CRE from 1850 to 2100, revealing a stability between 1850 and 1870, followed by a slight decline from the late 1870s to 2015. Notably, CERES data illustrate a significant decrease from 2000 to 2020. It should be noted that the sea ice coverage from CERES in Fig. 4a represents the snow and ice coverage denoted as SIC above, so it is not surprising that the values from CERES are higher that the CMIP6 outputs in Fig. 4a. When future scenarios of SSP 245 and SSP 585 are compared, a more pronounced decline in sea ice coverage is observed under SSP 585, in which the CRE exert a greater cooling effect than in SSP 245. Moreover, a significant positive relationship between sea ice coverage and CRE is observed (Fig. S4). These suggest that greater reductions in the Arctic sea ice correspond to more negative CRE (colder effect) in the future. Besides, as Fig. 4d shows, the multi-model averages of ∆CRE are –0.42 and –1.12 W/m^2^/decade during 1850–70 and 1871–2015, whereas they drop to –3.47 and –5.99 W/m^2^/decade in SSP 245 and SSP 585 at the TOA. At the SFC, there are similar patterns of ∆CRE to those at the TOA with smaller magnitudes, i.e. ∆CRE at the SFC are –0.07 and –0.23 W/m^2^/decade during 1850–70 and 1871–2015, and –0.64 and 1.08 W/m^2^/decade in SSP 245 and SSP 585. In historical, current and future periods, when there is a decline in the sea ice coverage or the SA, the CRE tend to be more negative to slow down the melting of the sea ice or the decrease in the SA. The greater the loss of sea ice or snow in the Arctic, the stronger the short-wave radiative cooling effect exerted by clouds—a phenomenon corroborated by both CMIP6 models and CERES data.

## DISCUSSION AND CONCLUSIONS

Clouds and the SA are critical in maintaining the energy balance of Earth—a significance that is heightened by global warming and Arctic Amplification. With the snow/ice melting in the Arctic, it is important to understand how snow/ice features affect cloud radiative processes and vice versa, and how they collectively participate in Arctic Amplification. Previous studies have explored these issues. For example, Alkama *et al.* [35] found that 66 ± 2% of this change in the net cloud radiative effect is due to the reduction in the SA and the remaining 34 ± 1% is due to an increase in cloud cover and optical thickness over polar regions. Loeb *et al.* [36] suggest that most of the variability in both the reflected short-wave (SW) TOA flux and the net downward SW surface flux anomalies is explained by variations in sea ice and CF alone over the Arctic Ocean. Some previous studies found that the short-wave CRE can be positive (warming effect) over a high SA, which implies that, with the SA declining, the warming effect could weaken and even turn out to be negative (cooling effect) [37,38]. Note that this phenomenon is similar to the aerosol short-wave warming effect that is observed over highly reflective surfaces, in which the warming effect decreases as the SA declines [39–42]. However, the interaction between cloud and snow/ice via the radiative process is still rarely quantified.

To address the above questions, we analyse the time evolution of the SIC and investigate the impacts of the SIC on the CRE in the Arctic region from the historical period to the present day and to future scenarios. From the perspective of satellites, the average SIC and SA values in the region of 60°N to 90°N significantly decreased from 2000 to 2020, with trends of –0.016/decade and –0.022/decade, respectively. The SIC and SA values show positive linear relationships in each grid such that a 1% change in the SIC causes an average 0.57% change in the SA, which indicates that the SIC and the SA have good spatio-temporal consistency. The decrease in the SIC is about –0.058/decade in these grids, with significant trends in both the SA and the SIC (*P* < 0.05) and *R*^2^ > 0.8, resulting in ∆CRE at the TOA and the SFC reaching –1.25 ± 0.49 and –0.21 ± 0.2 W/m^2^/decade, respectively. The ${\mathrm{\Delta }}\mathcal{R}$ is mainly located in the Beaufort, Chukchi, East Siberian, Laptev and Kara Seas, and in parts of the Greenland Sea, with the average being 3.45 cm/year. ∆CRE reaches the most negative value in June: –10.37 ± 0.59 and –2.67 ± 1.14 W/m^2^/decade at the TOA and SFC, with a corresponding value of ${\mathrm{\Delta }}\mathcal{R}$ of –2.27 ± 0.96 cm/year at the same time. From the perspective of state-of-the-art climate models of CMIP6, sea ice coverage remained stable during 1850–70 and showed a slight decline from 1870 to 2015 whereas, after 2015, it dramatically fell in both the SSP 245 and the SSP 585 scenarios. With the SIC melting, the CRE and ∆CRE tend to be more negative from the historical period to the present day and to future scenarios. The collaborative evolution of sea ice coverage and CRE aligns with the findings from satellites, confirming that the higher the loss of snow/ice in the Arctic, the stronger the short-wave cooling effect exerted by clouds, which partly mitigates snow/ice melting. This is similar to a previous study which suggested that years with less sea ice and a larger net surface radiative flux show a more negative cloud radiative effect [35], thus Arctic clouds can dampen the ice–albedo feedback mechanisms [43].

With the decreasing SA, traditional ice–albedo feedback mechanisms would predict an acceleration in ice and snow melt. However, as demonstrated in this study, clouds serve as a mitigating force within this acceleration, illustrating the self-regulating capabilities of Earth. However, the intensifying influence of anthropogenic activities on the climate, fostering its complex evolution, raises concerns about the persistence of such mitigation and the potential emergence of novel mechanisms, warranting further investigation.

## METHODS

### Data

The CERES project, as part of the Earth Observing System, provides satellite-based observations of the radiant energy of Earth [44]. The temporal scale of these observations ranges from hourly to monthly and the spatial scale ranges from 20 km to global observations. The CERES instruments on the Terra and Aqua satellites directly measure radiative fluxes, encompassing both reflected solar radiation and thermally emitted radiation, across a broad spectrum from ultraviolet to far-infrared wavelengths. In this study, we use the Synoptic Top of Atmosphere and Surface Fluxes and Clouds Level 3 product (SYN1deg Level 3 product), which includes short-wave fluxes under both clear-sky and cloudy-sky conditions at the TOA and SFC. Furthermore, the SYN1deg product offers the CF and COD data that are necessary for our study. By using the MODIS cloud-masking algorithm, MODIS pixels can be identified as clear or cloudy. In the SYN1deg Level 3 product, the CF is then determined as the fraction of MODIS pixels identified as cloudy out of the total pixels (cloudy plus clear sky). The COD is derived from the 0.65-micrometer channel data of the MODIS. At night, the COD is determined by using the MODIS infrared channel. The temporal span of the radiation and cloud properties data extends from March 2000 to February 2020 and the spatial span is from 60°N to 90°N. The time resolution of these data is monthly and the spatial resolution is 1° × 1°. SA retrieval varies between land and ocean, and depends on the sky conditions. For land, it is derived from the clear-sky TOA albedo based on CERES measurements and the Fu–Liou radiative transfer model [45,46]. For ocean, it is obtained by using a validated coupled ocean–atmosphere radiative transfer model to create an ocean albedo look-up table by considering factors such as the solar zenith angle, wind speed, cloud, aerosol optical depth, chlorophyll concentration and sea ice conditions [47]. The MODIS cloud mask significantly enhances thin cirrus detection, low cloud discrimination and cloud identification over bright snow/ice surfaces, improving SA retrieval accuracy [48]. The integration of land and ocean SA measurements by CERES SYN1deg provides a quality-ensured monthly SA dataset.

SIC data come from the Special Sensor Microwave Imager/Sounder instrument on the United States Defense Meteorological Satellite Program satellites and are provided in daily 25 km × 25 km grids. The SIC data can be download from the CERES website, so the *y*-axis in Fig. 4a named sea ice coverage refers to the SIC in CERES, similarly to the *x*-axis in Fig. S4. Sea ice thickness data are obtained from the Advanced Very High Resolution Radiometer on board the Polar Pathfinder, available in 25 km × 25 km grids every 12 hours [49]. Monthly data for SIC and sea ice thickness are used in this study, spanning from March 2000 to February 2020 at 60°N to 90°N. The SIC and sea ice thickness data are interpolated into a 1° × 1° spatial grid to align with the CERES data. In this study, the datasets above cover the same temporal and spatial range, and have the same resolution. It is important to distinguish this from the sea ice concentration (also abbreviated as SIC in other contexts), which is a measurement of the amount of sea ice in a given ocean area, usually described as a percentage. To avoid confusion, in this paper, ‘SIC’ exclusively refers to snow/ice coverage whereas sea ice concentration will be explicitly stated where relevant.

Nine state-of-the-art global climate models from the Coupled Model Inter-comparison Project Phase 6 (CMIP6) archive are selected and shown in Table S1. They provide monthly sea ice coverage and short-wave radiation under both clear-sky and all-sky conditions, with the SA being the ratio of the upward short-wave radiation and the downward short-wave radiation in the clear sky at the surface. The outputs from CMIP6 include the historical runs from 1850 to 2015 and two SSPs from 2015 to 2100. SSP 245 represents the middle-of-the-road scenarios with moderate population and economic growth with an end-of-the-century radiative forcing of 4.5 W/m^2^ whereas SSP 585 represents the business-as-usual scenarios with strong economic growth relying on fossil fuels with and end-of-the-century radiative forcing of 8.5 W/m^2^. The first ensemble member (r1i1p1f1) is used from each model and the native resolution of each model is kept before each variable is averaged over the Arctic (>60°N). We chose nine CMIP6 models that provided a robust representation of key climate features, particularly sea ice variability patterns, as these models exhibit strong similarities in their simulated sea ice trends and overall temporal evolution. Previous studies [50] have shown that CMIP6 models are generally capable of simulating multidecadal changes in Arctic sea ice since the mid-twentieth century. The selected models span a range of types and resolutions, ensuring that they are representative of the broader CMIP6 ensemble, with consistent results in sea ice trends across different models. Our study focuses on long-term trends and their impact on the CRE in Fig. 4 rather than a comprehensive evaluation of all CMIP6 models, meaning that the chosen subset is sufficient for our research objectives.

### The contribution of SIC to CRE

In each grid, the linear relationship between the SIC and the SA is established to estimate the contribution of the SIC to the SA, as shown in Equation (1):


(1) \begin{eqnarray*}
SA = a*SIC + b + \varepsilon,
\end{eqnarray*}

where *a* and *b* are empirical parameters. Specifically, *b* represents the SA value when SIC equals 0 and $\varepsilon $ represents the error term or residual. Only grids in which both the SIC and the SA show significant trends, satisfying *P* < 0.05 and *R*² > 0.8, are selected for further analysis. We use Equation (1) and SIC data to estimate the contribution of the SIC to the SA and further quantify the impact of the SIC and the CRE.

The TOA albedo (*R*) and the atmospheric system transmittance (*T*) as a function of SA are derived, which aids in determining the contributions of the surface and atmosphere to reflected short-wave radiation at the TOA, net downward SW radiative flux ate the SFC and the CRE [25,36]. The cloud contributions to the TOA albedo are determined by recent studies [26,27]. This study adopts a similar methodology to quantify the impact of the SA on the CRE at both the TOA and the SFC. Assuming the atmosphere is a single, uniform layer with a given reflectance (*r*) and transmittance (*t*), *r* and *t* can be derived from the short-wave radiative flux at the TOA and surface from CERES and climate models [28,51], with the details shown by Equations (S4)–(S7). *R* and *T* can be described by using Equations (2) and (3), respectively [52]:


(2) \begin{eqnarray*}
R = r + \frac{{SA\ {\mathrm{*}}\ {t^2}}}{{1 - SA\ {\mathrm{*}}\ r}}
\end{eqnarray*}

(3) \begin{eqnarray*}
T = \frac{t}{{1 - SA\ {\mathrm{*}}\ r}}
\end{eqnarray*}

It should be noted that Equations (S4)–(S7) and Equations (2) and (3) are valid under both the all-sky and the clear-sky conditions. The relationship of the CRE at the TOA and the SFC with respect to the SA can be established in a grid. For each selected grid, this relationship can be represented as shown in Equations (4) and (5). It is assumed that the scattering and absorption by molecules, aerosols and cloud are independent when calculating the CRE in Equations (4) and (5), which is reasonable due to the low aerosol loading in the Arctic:


(4) \begin{eqnarray*}
CR{E_{toa}} = {\mathrm{F}}_{TOA}^ \downarrow {\mathrm{*}}\left( {{R_{clr}} - {R_{all}}} \right){\mathrm{*}}\ CF,
\end{eqnarray*}

(5) \begin{eqnarray*}
CR{E_{sfc}} = {\mathrm{F}}_{TOA}^ \downarrow {\mathrm{*}}\left( {{T_{all}} - {T_{clr}}} \right){\mathrm{*}}\left( {1 - {\mathrm{SA}}} \right){\mathrm{*}}\ CF,
\end{eqnarray*}

where *CRE_toa_* and *CRE_sfc_* represent the CRE at the TOA and the SFC, respectively. CRE specifically refers to the short-wave cloud radiative effects whereas the total or long-wave cloud radiative effects will be explicitly mentioned where applicable. The subscripts of *all* and *clr* in Equations (4) and (5) represent the all-sky and clear-sky conditions, respectively. *R_clr_* and *R_all_* represent the TOA albedo under the clear-sky and all-sky conditions, respectively, whereas *T_clr_* and *T_all_* represent the atmospheric system transmittance under the clear-sky and all-sky conditions, respectively. ${\mathrm{F}}_{TOA}^ \downarrow $ represents the downward short-wave radiative flux at the TOA and *CF* is the cloud fraction. It should be noted that, during the freezing period, the SIC is close to 100%, which could lead to a high correlation between the SIC and the SA, potentially skewing the linear analysis. However, during the freezing period, short-wave radiation is minimal and thus the data for the SA are often missing (NaN). As the CRE are influenced by both the SIC and the SA, the absence of SA data automatically excludes these periods from the fitting process, ensuring that they do not impact the analysis significantly. Additionally, as the focus of our study is on regions in which the SIC and the SA show significant trends, the relationship between the SIC and the CRE is examined in the grids along the Beaufort, Chukchi, East Siberian, Laptev and Kara Seas, and parts of the Greenland Sea. In these regions, the SIC and the SA are not close to 100% and are thus more representative of the changes that we are studying. Therefore, the freezing period could not significantly affect the CRE–SIC–SA relationship in the regions of significant change.

The radiative energy depends strongly on the covariance between clouds and the surface. As Arctic snow and ice vary, the cloud properties are expected to respond, which in turn affects the evolution of snow and ice and modifies the SA [35,53,54]. To isolate the impact of the variation in clouds on the CRE and thus determine the change in the SA (${\mathrm{\Delta }}SA$) contribution to the change in the CRE (${\mathrm{\Delta }}CRE$) at the TOA (${\mathrm{\Delta }}CR{E_{toa}}$) and the SFC (${\mathrm{\Delta }}CR{E_{sfc}}$) during a period of time, we evaluate Equations (6) and (7) by using average values of CF ($\overline {CF} $), ${\mathrm{F}}_{TOA}^ \downarrow $ ($\overline {{\mathrm{F}}_{TOA}^ \downarrow } $), transmittance ($\bar T$) and surface albedo ($\overline {{\mathrm{SA}}} $) over a period of time:


(6) \begin{eqnarray*}
{\mathrm{\Delta }}CR{E_{toa}} &=& \frac{{\partial CR{E_{toa}}}}{{\partial {\mathrm{SA}}}}{\mathrm{*\ \Delta }}SA = \frac{{\partial CR{E_{toa}}}}{{\partial {R_{clr}}}}\frac{{\partial {R_{clr}}}}{{\partial SA}}{\mathrm{*\ \Delta }}SA \\
&& + \frac{{\partial CR{E_{toa}}}}{{\partial {R_{all}}}}\frac{{\partial {R_{all}}}}{{\partial SA}}{\mathrm{*\ \Delta }}SA \\
&\!\!\!\!=\!\!& \overline {{\mathrm{F}}_{TOA}^ \downarrow } {\mathrm{*}}\left( {\frac{{\partial {R_{clr}}}}{{\partial SA}} - \frac{{\partial {R_{all}}}}{{\partial SA}}} \right)\!{\mathrm{*}\ } \overline {CF} {\mathrm{*\ \Delta }}SA,
\end{eqnarray*}

(7) \begin{eqnarray*}
{\mathrm{\Delta }}CR{E_{sfc}} &=& \frac{{\partial CR{E_{sfc}}}}{{\partial {\mathrm{SA}}}}\ {\mathrm{*\ \Delta }}SA \\
&=& \frac{{\partial CR{E_{sfc}}}}{{\partial {T_{clr}}}}\frac{{\partial {T_{clr}}}}{{\partial SA}}\ {\mathrm{*\ \Delta }}SA \\
&&+ \frac{{\partial CR{E_{sfc}}}}{{\partial {T_{all}}}}\frac{{\partial {T_{all}}}}{{\partial SA}}\ {\mathrm{*\ \Delta }}SA \\
&=& \overline {{\mathrm{F}}_{TOA}^ \downarrow } {\mathrm{*}}\left\{ {\left( {\frac{{\partial {T_{all}}}}{{\partial SA}} - \frac{{\partial {T_{clr}}}}{{\partial SA}}} \right){\mathrm{*}}\left( {1 - \overline {{\mathrm{SA}}} } \right)}\right. \\
&&-\left. \vphantom{{\frac{{\partial {T_{all}}}}{{\partial SA}} - \frac{{\partial {T_{clr}}}}{{\partial SA}}}}{\left( {\overline {{T_{all}}} - \overline {{T_{clr}}} } \right)} \right\}{\mathrm{*}}\ \overline {CF} {\mathrm{*\ \Delta }}SA,
\end{eqnarray*}

where $\frac{{\partial R}}{{\partial SA}}$ and $\frac{{\partial T}}{{\partial SA}}$ are the changes in *R* and *T* that are caused by the SA under the all-sky or clear-sky conditions, which are shown in Equations (S8)–(S11). In grids in which both the SIC and the SA show significant trends, cases that satisfy *P* < 0.05 and *R*² > 0.8 are selected. Through linear fitting, the relationships between the SIC and the SA are established in all selected grids, thus ${\mathrm{\Delta }}SA$ is determined by a change in the SIC ($\Delta SIC$). By combining linear relationships with Equations (6) and (7), the ${\mathrm{\Delta }}SIC$ contributions to ${\mathrm{\Delta }}CR{E_{toa}}$ and ${\mathrm{\Delta }}CR{E_{sfc}}$ are determined in all selected grids during a period of time. Besides, the total change in the CRE during the same period is denoted as ${\mathrm{\Delta }}CR{E_{\textit{total}}}$ and the term ‘${\mathrm{\Delta }}CR{E_{\textit{total}}} - {\mathrm{\Delta }}CRE$’ represents the change in the CRE due to other factors besides the SA, as shown in Fig. S2.

The sea ice melting rate change (${\mathrm{\Delta }}\mathcal{R}$) due to a radiative flux change (${\mathrm{\Delta }}F$) can be estimated by using Equation (8), similarly to previous studies [55,56]. This is referred to as a sea ice growth model—a numerical or analytical framework that is designed to simulate sea ice variations by accounting for thermodynamic processes such as heat/latent fluxes and radiative energy flux:


(8) \begin{eqnarray*}
{\mathrm{\Delta }}\mathcal{R} = \frac{{{\mathrm{\Delta }}F}}{{\rho *L}}*\Delta t,
\end{eqnarray*}

where ${\mathrm{\Delta }}F$ can be ‘${\mathrm{\Delta }}CR{E_{sfc}}$’ or ‘${\mathrm{\Delta }}CR{E_{\textit{total}}} - {\mathrm{\Delta }}CRE$’ at the SFC, $\rho $ is the density of the ice (917 kg/m³) and *L* is the ice latent heat coefficient (333.4 kJ/kg). In this study, the time step ∆*t* is a decade aligning with the change in the SIC and the CRE. A more negative value of ${\mathrm{\Delta }}\mathcal{R}$ means that the decline in sea ice melting can be slowed down more by enhancing the short-wave cooling effect of the clouds. To estimate ${\mathrm{\Delta }}\mathcal{R}$ based on Equation (8), we selected grids in which the SIC and the SA exhibited significant trends and the relationship between the SIC and the SA in Equation (1) was statistically significant (*P* < 0.05). The selected grids primarily cover regions in which sea ice melting occurs, such as the Beaufort, Chukchi, East Siberian, Laptev and Kara Seas, as well as parts of the Greenland Sea.

This study primarily focuses on the impact of the SIC on the CRE and we also analyse how changes in the CRE affect ${\mathrm{\Delta }}\mathcal{R}$, which is the metric that is used to reflect the magnitude and sign of ${\mathrm{\Delta CRE}}$. This value of ${\mathrm{\Delta CRE}}$ represents the isolated contribution of cloud short-wave radiative effects on sea ice thickness over time and is not meant to suggest that cloud effects alone account for the entire reduction in sea ice thickness. Considering the interaction of various factors, the overall impact on sea ice thickness may differ.

### Propagating uncertainties in the CRE

The measurement errors of radiative flux in CERES is in the order of 1–10 W/m^2^ (∼O(1–10 W/m^2^)) per 1° × 1° monthly grid box and rapidly diminish to ∼O(0.001–0.01 W/m^2^) when averaging globally for a long time [57]. In the multi-year analysis of this study, measurement errors are represented by the standard deviation of the annual mean, as detailed in Table S2. In fact, systematic errors may pose more significant concerns [25]. Fortunately, by quantifying the propagating uncertainties from measurement errors, we found that they would not affect our conclusions (–1.25 ± 0.49 W/m^2^ for the CRE at the TOA and –0.21 ± 0.20 W/m^2^ at the SFC) that were drawn on the weakened snow and ice melting by the enhanced cloud radiative effect in the Arctic. Details of the propagating uncertainties in the CRE are provided in the material.

## Supplementary Material

nwaf116_Supplemental_File

## Data Availability

The SYN1deg Level 3 data and SIC data can be accessed from NASA-Clouds and the Earth's Radiant Energy System project at https://ceres.larc.nasa.gov/data/. CMIP6 data are available from the Earth System Grid Federation and can be downloaded from the US Department of Energy/Lawrence Livermore National Laboratory node at https://esgf-node.llnl.gov/projects/cmip6/. The land and sea boundary files used for generating figures are from the UCLA Geoportal. All code used in this work is available from the corresponding authors upon reasonable request.
