# Supplementary material for: Weakened snow and ice melting by enhanced cloud short-wave cooling effect in the Arctic
Source: Natl Sci Rev. 2025 Mar 27;12(6):nwaf116. doi: 10.1093/nsr/nwaf116 (PMC12051870; doi:10.1093/nsr/nwaf116)
Supplement: nwaf116_Supplemental_File [file nwaf116_supplemental_file.pdf]

Supplementary Material for

Weakened snow and ice melting by enhanced cloud short-wave cooling effect in the Arctic

Annan Chen[1], Chuanfeng Zhao[1*], Haotian Zhang[1], Yikun Yang[1], Jing Li[1], Yan Yu[1], Qinghong Zhang[1], Jiefeng Li[1]

[1] *Department of Atmospheric and Oceanic Sciences, School of Physics, Peking University, Beijing, China*

*Corresponding Author: Chuanfeng Zhao, 209

Email: cfzhao@pku.edu.cn

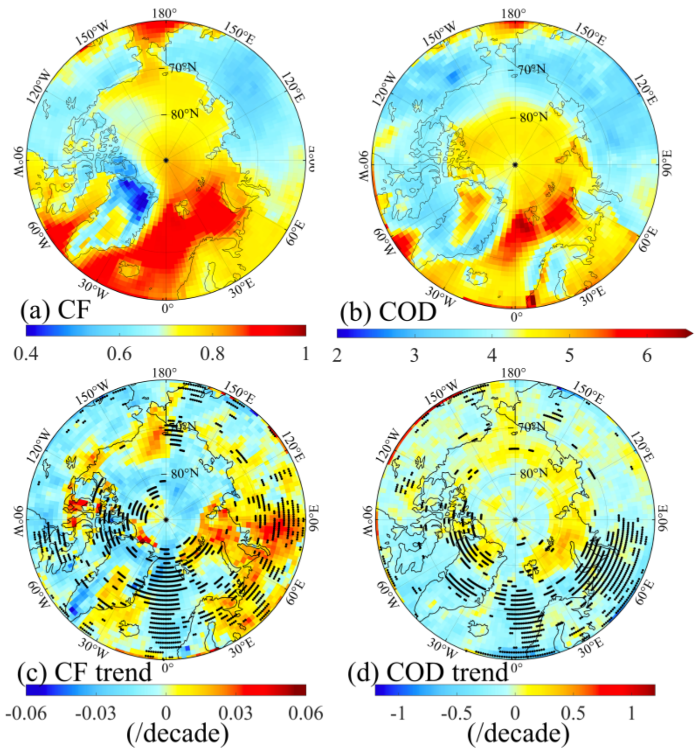

Fig. S1: Spatial and temporal distribution of cloud fraction (CF) and cloud optical depth (COD). (a) the spatial distribution of the multi-year average of CF from March 2000 to February 2020 in the 60 to 90°N; (b) similar to (a) but for COD; (c) and (d) show the long-term trends of CF and COD, respectively, with black dots indicating $P<0.01$.

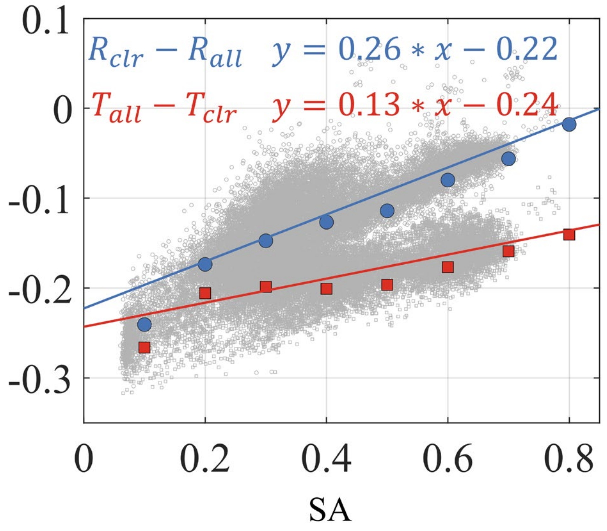

Fig. S2. Scatter plots and linear fittings between SA and $R_{clr} - R_{all}$ (gray circles and blue line, respectively), with blue circles representing the averages of $R_{clr} - R_{all}$ in each 0.1 SA interval, and between SA and $T_{all} - T_{clr}$ (gray squares and red line, respectively), with red squares representing the averages of $T_{all} - T_{clr}$ in each 0.1 SA interval. The selected grids are consistent with those in Fig. 2.

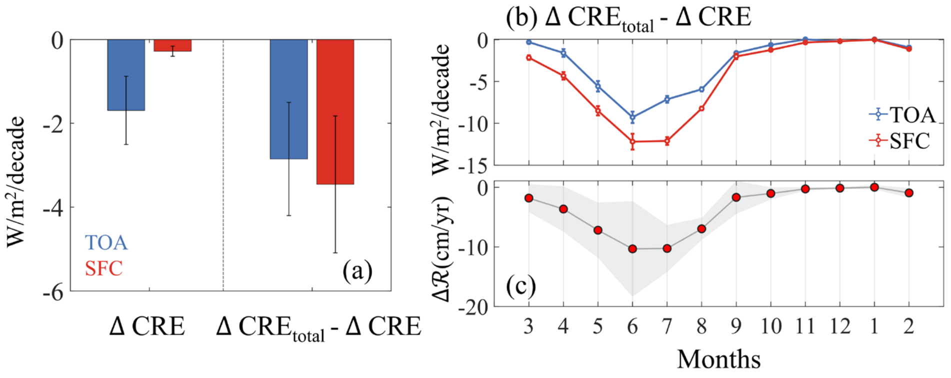

Fig. S3. (a) The change of CRE due to the multi-year change of SA (ΔCRE), and $\Delta CRE_{total} - \Delta CRE$, the error bars are the standard deviation; (b) the monthly variations of $\Delta CRE_{total} - \Delta CRE$ at the TOA and SFC; (c) the monthly variation of $\Delta\mathcal{R}$, with the shadow representing the standard deviation.

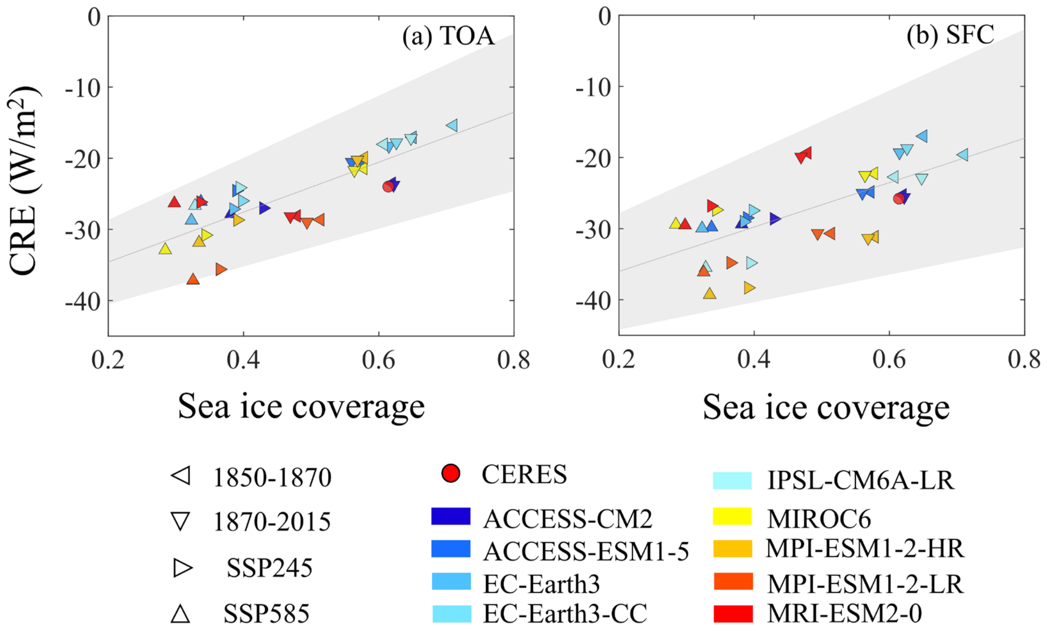

Fig S4. Relationships between cloud radiative effect (CRE) at the TOA (a), as well as at the SFC (b) and Sea ice coverage in different historical periods and two shared societal pathways (SSPs) from the outputs of 9 models participating in Coupled Model Inter-comparison Project Phase 6 (CMIP6). CMIP6 values from the historical, SSP 245 and SSP 585 runs are represented as color triangles (facing left for the 1850 to 1870 mean, down for the 1870 to 2015 mean, right for the SSP 245 and up for the SSP 585). Different colors represent different models, their linear fitting regressions and 95% confident fitting slope intervals are shown by gray lines and shading. The CERES are presented as red circles for the present day mean. The goodness of fit is 0.68 (a) and 0.46 (b).

Table S1. The information about climate models from CMIP6 used in this study

Models' name	Institutions	Grid (lon*lat)
ACCESS-CM2	Commonwealth Scientific and Industrial Research Organization	192*144
ACCESS-ESM1-5	Commonwealth Scientific and Industrial Research Organization	192*144
EC-Earth	EC-Earth Consortium	362*292
EC-Earth3-CC	EC-Earth Consortium	512*256
IPSL-CM6A-LR	Institute Pierre-Simon Laplace	144*143
MIROC6	Japan Agency for Marine-Earth Science and Technology	256*128
MPI-ESM1-2-LR	Max Planck Institute for Meteorology	192*96
MPI-ESM1-2-HR	Max Planck Institute for Meteorology	384*192
MRI-ESM2-0	Meteorological Research Institute	320*160

Table S2. The standard deviation (uncertainties) for yearly mean surface albedo, cloud fraction, downward short-wave radiation at the TOA, TOA reflectance and atmospheric system transmittance in the all-sky and clear-sky conditions.

Variables ($\mathrm{X_i}$)	Uncertainties ($\delta\mathrm{X_i}$)
Surface albedo (SA)	0.0035
Cloud fraction (CF)	0.0051
Downward short-wave radiation at the TOA ($F^{\downarrow}$)	0.082 W/m^2
TOA reflectance in the all-sky (R_{all})	0.0021
TOA reflectance in the clear-sky (R_{clr})	0.0028
Atmospheric system transmittance in the all-sky (T_{all})	0.0012
Atmospheric system transmittance in the clear-sky (T_{clr})	0.0017

The details about propagating uncertainties of CRE

Assuming variables are independent for simplicity and neglecting their covariances here. The variables uncertainties are listed in Table S2, which is used as input in the formulation of Gaussian error propagation, as shown in the Equation (S1):

$$\delta Y = \{\sum_{i=1}^{n}((\frac{\partial Y}{\partial X_i})^2 * \delta X_i{}^2)\}^{1/2} \qquad (S1)$$

where δY is the total uncertainty, X_i is the ith variable, δX_i represents the uncertainty of X_i, $\frac{\partial Y}{\partial X_i}$ is the partial derivatives of Y over X_i. The partial derivatives in Equation (S1) are evaluated using mean values X_j in the Arctic region, where $j \neq i$.

The formulation of Gaussian error propagation is applied in the function of CRE_{toa} (Equation (3)) and CRE_{sfc} (Equation (4)) in each grid to drive the uncertainties of CRE_{toa} (δCRE_{toa}) and CRE_{sfc} (δCRE_{sfc}), which are shown in the Equation (S2) and (S3):

$$\delta CRE_{toa} = \left((\frac{\partial CRE_{toa}}{\partial R_{all}})^2 * \delta R_{all}{}^2 + (\frac{\partial CRE_{toa}}{\partial R_{clr}})^2 * \delta R_{clr}{}^2 + (\frac{\partial CRE_{toa}}{\partial CF})^2 * \delta CF^2 + (\frac{\partial CRE_{toa}}{\partial F^{\downarrow}})^2 * \delta F^{\downarrow 2}\right)^{1/2} \qquad (S2)$$

$$\delta CRE_{sfc} = \left((\frac{\partial CRE_{sfc}}{\partial SA})^2 * \delta SA^2 + (\frac{\partial CRE_{sfc}}{\partial T_{all}})^2 * \delta T_{all}{}^2 + (\frac{\partial CRE_{sfc}}{\partial T_{clr}})^2 * \delta T_{clr}{}^2 + (\frac{\partial CRE_{sfc}}{\partial CF})^2 * \delta CF^2 + (\frac{\partial CRE_{sfc}}{\partial F^{\downarrow}})^2 * \delta F^{\downarrow 2}\right)^{1/2} \qquad (S3)$$

where δSA, δCF, $\delta F^{\downarrow}$, δR_{all}, δR_{clr}, δT_{all}, and δT_{clr} are the uncertainties of surface albedo, cloud fraction, downward short-wave radiation at the TOA, TOA reflectance and atmospheric system transmittance in the all-sky and clear-sky conditions, which are listed in Table S2.

The details about the derivations of atmospheric reflectance (r) and transmittance (t)

The upward short-wave radiative flux at TOA ($F_{TOA}^{\uparrow}$) is composed of the reflected by downward short-wave radiative flux at TOA ($F_{TOA}^{\downarrow}$) and transmitted by the upward short-wave radiative flux at SFC ($F_{sfc}^{\uparrow}$), as shown in Equation (S4); Similarly, the downward short-wave radiative flux at SFC ($F_{sfc}^{\downarrow}$) is composed of the transmitted by the downward short-wave radiative flux at TOA ($F_{TOA}^{\downarrow}$) and the reflected by upward short-wave radiative flux at SFC ($F_{sfc}^{\uparrow}$), as shown in Equation (S5).

$$F_{TOA}^{\uparrow} = r * F_{TOA}^{\downarrow} + t * F_{sfc}^{\uparrow} \quad \text{(S4)}$$

$$F_{sfc}^{\downarrow} = t * F_{TOA}^{\downarrow} + r * F_{sfc}^{\uparrow} \quad \text{(S5)}$$

Ignoring the difference of r in the direction (upward and downward) in Equations (S4) and (S5), as well as that of t, the r and t can be derived from Equations (S4) and (S5):

$$r = \frac{F_{TOA}^{\downarrow} * F_{TOA}^{\uparrow} - F_{sfc}^{\downarrow} * F_{sfc}^{\uparrow}}{(F_{TOA}^{\downarrow})^2 - (F_{sfc}^{\uparrow})^2} \quad \text{(S6)}$$

$$t = \frac{F_{TOA}^{\downarrow} * F_{sfc}^{\downarrow} - F_{TOA}^{\uparrow} * F_{sfc}^{\uparrow}}{(F_{TOA}^{\downarrow})^2 - (F_{sfc}^{\uparrow})^2} \quad \text{(S7)}$$

In Equations (S4) to (S7), $F_{TOA}^{\uparrow}$, $F_{TOA}^{\downarrow}$, $F_{sfc}^{\downarrow}$ and $F_{sfc}^{\uparrow}$ are provided by CERES SYN1deg data and climate models both in the clear-sky and all-sky conditions.

The determination of the changes in *R* and *T* caused by SA in the all-sky and clear-sky conditions

$$\frac{\partial R_{all}}{\partial SA} = \frac{\overline{t_{all}^2}}{(1-\overline{SA}*\overline{r_{all}})^2} \quad \text{(S8)}$$

$$\frac{\partial R_{clr}}{\partial SA} = \frac{\overline{t_{clr}^2}}{(1-\overline{SA}*\overline{r_{clr}})^2} \quad \text{(S9)}$$

$$\frac{\partial T_{all}}{\partial SA} = \frac{\overline{r_{all}}*\overline{t_{all}}}{(1-\overline{SA}*\overline{r_{all}})^2} \quad \text{(S10)}$$

$$\frac{\partial T_{clr}}{\partial SA} = \frac{\overline{r_{clr}}*\overline{t_{clr}}}{(1-\overline{SA}*\overline{r_{clr}})^2} \quad \text{(S11)}$$

The overline on the variables in the Equations (S8) to (S11) denotes the averaged value over a period of time.
